# Heme oxygenase/carbon monoxide system and cardiac conduction system

**DOI:** 10.4103/mgr.MEDGASRES-D-25-00195

**Published:** 2026-04-11

**Authors:** Vicki L. Mahan

**Affiliations:** 1 Department of Surgery, Queen Elizabeth Central Hospital, Blantyre, Malawi; 2 Drexel University Medical School, Philadelphia, PA, USA

**Keywords:** atrioventricular node, bundle branch, carbon monoxide, cardiac conduction system, gasotransmitter, heme oxygenase, His bundle, HO/CO system, peripheral conduction system, Purkinje cell, sinoatrial node

## Abstract

**Facts**

1. The heme oxygenase/carbon monoxide system is required for cardiomyocyte development.
2. Developing cardiomyocytes experience fluctuating oxygen levels. Heme oxygenase-1, which is induced by hypoxia, protects the cardiomyocytes.
3. Heme oxygenase activity and its products influence biochemical processes through transcription factors that are needed for cardiac development.
4. The cells of the cardiac conduction system are specialized cardiomyocytes that are modified for electrical signaling, not contraction.
5. Carbon monoxide modulates vascular tone and inflammation, and protects against oxidative stress. It is required to maintain the health and function of the cardiac conduction system.

**Open Questions**

1. How do the effects of the heme oxygenase/carbon monoxide system differ between contractile and cardiac conduction system cardiomyocytes?
2. Are the effects of the heme oxygenase/carbon monoxide system different for the different structures of the cardiac conduction system (sinoatrial node, atrioventricular node, His bundle, bundle branches, and Purkinje fibers)?
3. Is carbon monoxide a biomarker for cardiac conduction system abnormalities? If so, can it be used for risk stratification?
4. How can the heme oxygenase/carbon monoxide system be used to treat cardiac conduction system abnormalities?
5. What cardiac conduction abnormalities occur if heme oxygenase-1 is lacking during in utero development?

The heme oxygenase/carbon monoxide system is well-established as a critical regulator of contractile cardiomyocyte development. Although the heme oxygenase/carbon monoxide system is known to have protective effects against arrhythmias by reducing oxidative stress and preserving mitochondrial function in general myocardium, its specific impact on components of the cardiac conduction system, such as the sinoatrial and atrioventricular nodes, is unclear. On the one hand, the antioxidant and anti-inflammatory properties of the heme oxygenase/carbon monoxide system are believed to help maintain normal electrical conduction and prevent arrhythmias. On the other hand, pathological conditions such as age-related sick sinus syndrome have been linked to dysfunction of the heme oxygenase/carbon monoxide system, even though carbon monoxide is detrimental at toxic levels. This review synthesizes the current evidence on the dual nature of the heme oxygenase/carbon monoxide system in the cardiac conduction system. This review highlights the critical need for further investigation of the heme oxygenase/carbon monoxide system’s physiological roles and therapeutic potential for conduction pathologies.

## Introduction

**Figure 1:**
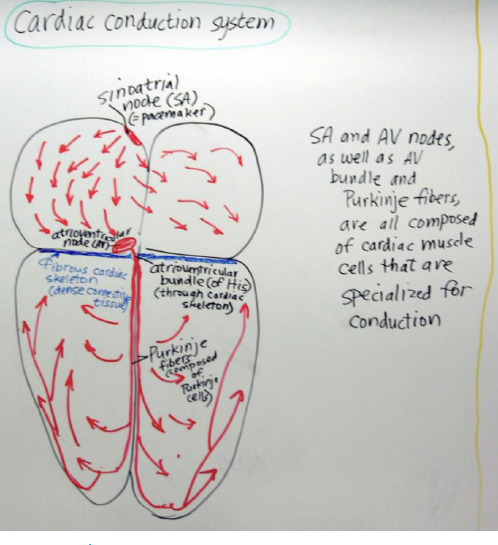
A hand drawn sketch of cardiac conduction system. The cardiac impulse originates in the sinoatrial node and travels to both atria and the atrioventricular node. The impulse continues through the atrioventricular node and fibrous body to the His bundle, the bundle branches, and the Purkinje fibers before innervating the cardiomyocytes that contract. Created by A. Kent Christensen under a Creative Commons license: BY-SA. AV: Atrioventricular; SA: sinoatrial.

Origin of the human heartbeat revolves around two different hypotheses: the neurogenic theory, where nerves are postulated to be responsible for contractile timing, and the myogenic theory, where a portion of the heart is capable of beating spontaneously and rhythmically.^1^ Anatomic discoveries and experiments helped to establish that the heart’s innate ability to generate electrical impulses is responsible for the heartbeat. Martin Flack, a medical student, discovered the sinoatrial node (SAN), the site of the origin of the heartbeat.^2^ Driven by the movement of ions across heart cell membranes, studies have determined that changes in intracellular Ca^2+^ result in cardiac conduction and contraction.^3^,^4^ The “coupled clock system” of the SAN, a complex pathway in the pacemaker cells of the heart, results in the origin of the initial electrical impulse. Two “clocks” work together to generate electrical impulses that determine the heart rate. These two “clocks” are the membrane voltage clock, which is based on the electrical activity of the cell membrane and determined by ion channels, and the calcium clock, which is determined by rhythmic release of calcium ions from the sarcoplasmic reticulum in the cell and affects the membrane potential.^5^,^6^ These cardiac impulses originate in the SAN and can be conducted by all cardiomyocytes in the heart (**Figure 1**).

The cardiac impulse that originates in the SAN is conducted through the cardiac conduction system (CCS) to the peripherally located contractile cardiomyocytes.^7^ Coordination and timing of cardiac contraction depend on the electrical structure of the heart, formed by specialized cardiomyocytes that consists of the SAN, atrioventricular node (AVN), His bundle, bundle branches, and peripheral conduction system. The specialized cells and the atrial and ventricular working myocardium are derived from shared precursor cells that diverge during growth and development. Specific transcription factors activate a pacemaker gene program and suppress the atrial and ventricular working myocardium gene program in the CCS. Functionally, the CCS is classified into slow-conducting structures (AVN and SAN) and fast-conducting ventricular conduction system, including Purkinje system (part of peripheral conduction system), His bundle, and left and right bundle branches.^8^ During development, the His bundle and bundle branches have pacemaker activities but also acquire fast-conducting properties. The Purkinje system, derived from the embryo’s trabecular myocardium (network of specialized cells in the ventricles that rapidly transmit the electrical impulses), acquires fast-conducting properties from the onset.^9^ These electrical forces affect the morphology of the cardiac chambers.^10^

The posterior cardiogenic progenitor cells from the second heart field give rise to the CCS.^11^ Impulses, normally generated by pacemaker cardiomyocytes of the SAN, travel through the atria to the AVN/atrioventricular bundle, then to the left and right bundle branches, and finally to the more peripheral conduction system.^12^ Impulses are then transmitted from the peripheral conduction system to the contractile cardiomyocytes, resulting in coordinated contraction of the heart. The SAN anlage can be identified morphologically in the mouse at about embryonic day 10. Development is within the sinus venosus next to the embryonic right atrial wall.^13^ The SAN is a structure of connective tissue with a small number of specialized cells that are electrically connected. Diastolic depolarization is the main determinant of the pacemaker’s rate. The pacemaker cells do not have a stable resting potential, but rather have a slowly rising depolarization, which includes the end of the action potential repolarization to the upstroke of the next action potential.^14^ The funny current (If) is the main contributor to diastolic depolarization and is an inward Na^+^/K^+^ current that is acted on by cyclic adenosine monophosphate and responds to hyperpolarization.^15^

The heme oxygenase (HO)/carbon monoxide (CO) system is crucial for cardiomyocyte growth, development, and maturation and provides cardiomyocyte protective effects. HO reduces oxidative stress, inhibits apoptosis, has anti-oxidant and anti-inflammatory effects, reduces senescence, inhibits hypertrophy, and prevents fibrosis. The specialized cardiomyocytes of the CCS and contractile cardiomyocytes originate from the same group of progenitor cells during embryonic development. The HO/CO system affects contractile cardiomyocytes, but specific effects on the CCS have not been studied. However, in mice with aging-induced sick sinus syndrome, *Yixin*-*Fumai* granules (activate the nuclear factor erythroid 2-related factor 2 (Nrf2)/HO-1 pathway and increase the HO expression), increase heart rate, alleviate fibrosis, reduce apoptotic rate and reactive oxygen species content, and promote hyperpolarization-activated cyclic nucleotide-gated channel 4 expression in the SAN, thereby inhibiting aging-induced sick sinus syndrome. The hyperpolarization-activated cyclic nucleotide-gated channel 4 channel allows sodium and potassium ions to flow into cells of the SAN.^16^,^17^ This suggests that the HO/CO system affects the CCS. Effects on the CCS may be different from those seen on contractile cardiomyocytes, vary with the different structures, and change as the specialized cardiomyocytes grow and mature. HO is required for both the physiological and pathological cardiac functions of the heart.^18^ Its roles on contractile cardiomyocytes continue to be evaluated and recommendations made with regard to possible HO/CO system therapies. However, little is known about the HO/CO system effects on the specialized cardiomyocytes of the CCS. This review will begin to address the role of the HO/CO system on the different structures of the CCS and its therapeutic implications.

## Search Strategy

I searched PubMed, MEDLINE, Google Scholar, HubMed, Embase, and the Cochrane Library with the search terms of cardiac conduction system, sinoatrial node, cardiac progenitor cells, atrioventricular node, hisbundle, bundle of His, bundle branches, Purkinje fibers, peripheral conduction system, gasotransmitters, heme oxygenase, HO, carbon monoxide, and CO from 1970 to 2025. Inclusion criteria included CCS and its development, effects of HO/CO system on development and changes of the CCS, SAN, AVN, His bundle, left and right bundle branches, Purkinje system, HO/CO system, and stages of growth and development of the CCS.

## General Anatomy and Function of the Human Conduction System of the Heart

**Figure 2:**
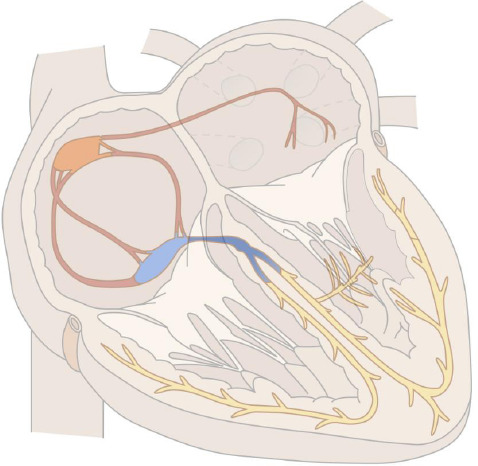
The cardiac conduction system. The sinoatrial and atrioventricular nodes are located in the right atrium. Impulses originating in the sinoatrial node traverse several pathways to reach the atrioventricular node. Bachmann’s bundle is a band of specialized muscle fibers in the heart that serves as the main pathway for electrical impulses to travel from the right atrium to the left atrium. Impulses are transmitted through the atrioventricular node to the His bundle and then the bundle branches. The left bundle branch bifurcates into the anterior and posterior bundles. From the bundle branches, the impulse reaches the Purkinje fibers and then the cardiomyocytes needed for myocardial contraction. Modified version by uploader of this file. Original by YitzhakNatKeanu Dölle, CC BY-SA 4.0, via Wikimedia Commons.

The CCS consists of four structures: the SAN, the AV node, the atrioventricular bundle, and the Purkinje fibers/peripheral conduction system. These structures are made up of modified cardiomyocytes that are specialized for conduction. The SAN (pacemaker of the heart) is located near the opening of the superior vena cava into the right atrium. Impulses originating in the SAN are conducted from right to left across both atria. As the atria depolarize, the AVN (located in the right atrium in the triangle of Koch, conduction pathway between atria and ventricles) gathers the impulse and the speed of the impulse is reduced. The impulses then travel down the atrioventricular bundle which transmits the impulse to two pathways on the interventricular ventricular septum (speed of transmitted impulse increases), and then (about half way down interventricular septum) the impulse is transmitted through Purkinje fibers which extend to all areas of both ventricles (**Figure 2**). This results in depolarization of the myocardium followed by myocardial contraction.

**Figure 3:**
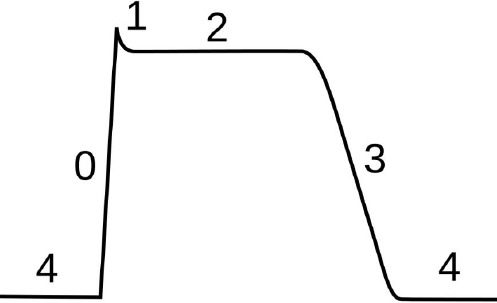
Normal action potential. An electrical signal, a normal cardiac action potential, involves five phases. Phase 0 is the rapid depolarization/upstroke phase. Early repolarization occurs during Phase 1. Phase 2, the plateau phase, reflects a relatively positive level for a period of time. Phase 3 is the repolarization phase. Phase 4 is the resting membrane potential, where the cell is polarized. CC BY-SA 3.0, via Wikimedia Commons.

**Figure 4:**
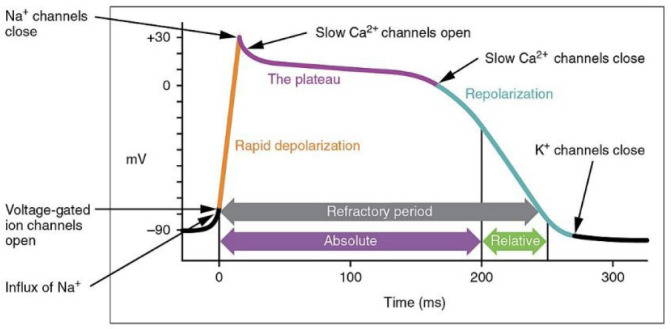
Ion movement in normal cardiac action potential. Specific ion channel activity occurs during each of the five phases of the normal cardiac action potential. During Phase 0 (rapid depolarization), Voltage-gated Na^+^ channels rapidly open in response to a stimulus, which allows the influx of Na^+^ into the cell. The membrane potential becomes less negative during this phase. Early repolarization, Phase 1, occurs when the fast Na^+^ channels are inactivated. This stops the inward Na^+^ current. Transient outward currents result in an initial repolarization. Phase 2, the plateau phase, occurs when L-type Ca^2+^ channels open, allowing an inward current of Ca^2+^ into the cell. This is balanced by outward K^+^ currents. The effect triggers muscle contraction. L-type Ca^2+^ channels close during Phase 3 (repolarization). There is a large efflux of K^+^ through outward-rectifying potassium channels, resulting in the return of the membrane potential to negative values. The resting membrane potential (Phase 4) is mainly determined by the inwardly rectifying potassium channels. The Na^+^/K^+^-ATPase pump and other ion exchangers are required to maintain the Na^+^ and K^+^ gradient across the membrane. OpenStax College, CC BY 3.0, via Wikimedia Commons.

**Figure 5:**
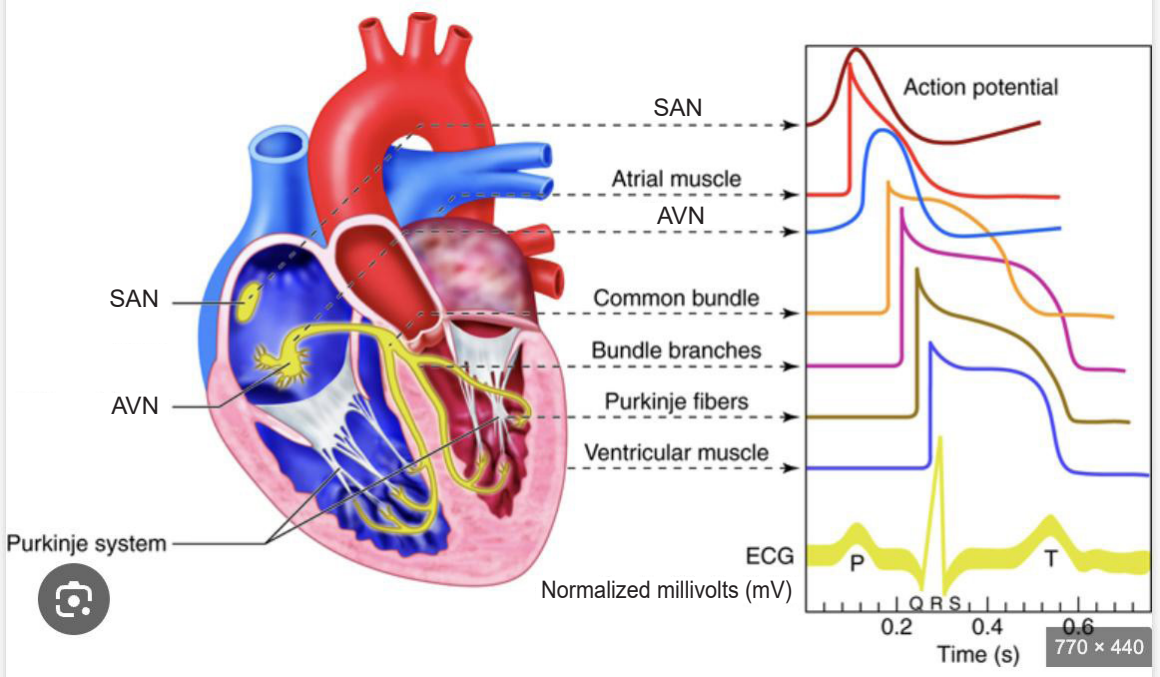
Differences in action potentials of pacemaker cells and contractile cardiomyocytes. The two main types of cardiac action potentials are slow/brief (seen in the SAN and AVN) and fast/long (seen in His bundle, bundle branches, Purkinje fibers, and contractile cardiomyocytes). The primary differences are their conduction velocity, mechanism of depolarization, and resting membrane potential. Created 2025 Open Learning Initiative, Carnegie Mellon University. Licensed under CC BY-NC-SA 4.0. AVN: atrioventricular node; ECG: electrocardiogram; SAN: sinoatrial node.

Normal action potentials, rapid transient changes in membrane voltage used to transmit signals, usually involve depolarization to a positive peak followed by repolarization back to its resting potential with a short period of hyperpolarization (**Figure 3**). Sodium, potassium, and calcium channels are required for its effects (**Figure 4**). Cardiac action potentials are generated by pacemaker cells with slow-response action potentials (seen in SAN and AVN) and contractile myocytes with fast-response action potentials (seen in normal atrial and ventricular cardiomyocytes and Purkinje fibers).^24^,^25^,^26^ Fast-response action potentials have five distinct phases with rapid depolarization followed by a long plateau. Slow-response action potentials seen in the SAN and AVN lack phases 1 and 2 (**Table 1**). The action potentials of the different structures of the CCS are different and each has its own unique identifying features (**Figure 5**).

**Table 1:** Differences in phases of action potential between pacemaker cells and contractile cardiomyocytes

Phase of cardiac action potential	Pacemaker cells	Contractile cardiomyocytes
0	Upstroke occurs. Rapid depolarization occurs in other cells, but in pacemaker cells Phase 0 is slower and more gradual	Voltage-gate sodium channels open, causing a rapid influx of sodium and a shift in membrane potential to a positive voltage range. This phase is important for the rapid promulgation of the cardiac impulse
1	Absent in pacemaker cells	Sodium channels inactivate and a small number of potassium channels open. This phase sets the stage for the next phase of the action potential
2	Absent in pacemaker cells	Voltage-gate calcium channels open, allowing calcium to enter the cell. This is the longest phase of the action potential and is unique to excitable cells
3	Calcium channels are closed and potassium channels are opened, allowing potassium efflux. This process enables the membrane to revert to its resting state	More potassium channels open
4	Membrane potential slowly depolarizes from the resting state towards threshold. This results from a combination of sodium influx and potassium efflux. This is the key to the pacemaker activity	The sodium/potassium ATPase pump exchanges three sodium ions for two potassium ions, maintaining the negative intracellular potential

The action potentials (APs) generated by the pacemaker cells in the SAN and conducted by the CCS travel through gap junctions to the contractile cardiomyocytes. These specialized channels, formed by connexin proteins (three main subtypes in the heart – connexin (Cx)40, Cx43, and Cx45), are important for the movement of ions and small molecules between cells. Primarily located in intercalated discs and critical to cell-to-cell transmission of cardiac impulses, gap junctions allow the electrical signals to propagate from the SAN through the atria, atrioventricular node, His bundle, and its bundle branches, ultimately reaching the Purkinje cells to trigger myocardial depolarization and contraction. Transmission of APs from one cell to the next results in electrical coupling (passive spread of charge between cells). Depolarization (electrical charge inside a cell becomes less positive) of the membrane occurs along the sarcolemma and into the t-tubules. The voltage-sensitive dihydropyridine receptors on the t-tubules open L-type calcium channels (allow calcium ions to enter cells during the plateau phase).^27^ The calcium flux allows activation of the ryanodine receptor (RYR) – a calcium release channel in the sarcoplasmic reticulum membrane. Dihydropyridine receptors and RYRs (positioned on opposite sides of junctional gap separating external and membrane systems) result in functional/structural coupling between the proteins (play a key role in excitation-contraction coupling).^28^ The cell is flooded with calcium, resulting in regulatory proteins detaching from actin, which allows myosin to bind. Calcium ions bind to troponin C and interact with tropomyosin to unblock active sites between actin and the myosin filament. These cross bridges provide the power stroke for contraction, releasing the remaining calcium from storage in the sarcoplasmic reticulum to complete the process.^29^,^30^

## Heme Oxygenase/Carbon Monoxide System and Sinoatrial Node

**Figure 6:**
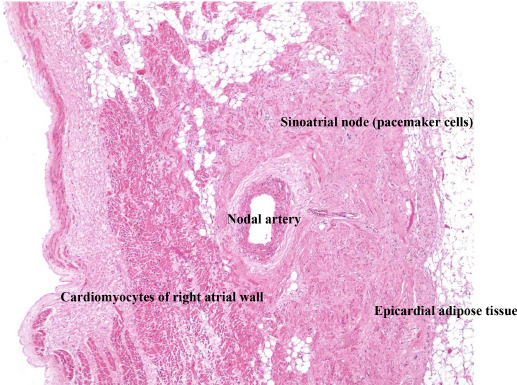
Histology of sinoatrial node. Sinoatrial node cardiomyocytes are stained less intensely than contractile cardiomyocytes. The nodal artery is seen in the center surrounded by the sinoatrial node. The sinoatrial node abuts the right atrial cardiac myocytes. Nephron, CC BY-SA 3.0, via Wikimedia Commons.

From the center of the SAN to the periphery, there is a gradient of AP shape, pacemaking, connexin expression, Ca^2+^ handling, ionic current densities, cell size, and myofilament density.^31^ The cardiac pacemaker cells (CPCs) are nested in an extracellular-rich matrix and are termed the SAN (**Figure 6**). Microarchitecture is atypical – diversity and specialization of the extracellular matrix are present in the adult SAN. While the HO/CO system is known to be important to the development of the embryo/fetus in utero, research is at the newborn stage with regards to the effects of the system on the growth and development of the conceptus and data regarding the effects on development and effects of the system on the SAN in utero are not available.

Henley et al.^32^ determined that there is a developmental window during which mature CPCs’ cellular architecture is seen, which coincides with the expression of genes that are associated with extracellular encapsulation. The extracellular matrix enhances expression of genes specific to the pacemaker (and their automaticity).^33^ Hyaluronic acid-based proteoglycan-rich extracellular matrix surrounds CPCs during SAN morphogenesis – the mechanical properties of this region are not as vigorous as neighboring atrial wall motion. Subjection of CPCs to substrate stiffness greater than what is normally present results in loss of normal electrochemical oscillation and disruption of ion channel localization.^34^ CPCs may be located in an environment that protects the structure from forces normally experienced by myocardial cells. SAN function depends on this complex architecture and functioning of gap junctions and ion channels that are different from the atrial working myocardium. Mechanics are crucial to embryonic CPC function and determine the need for maturation of embryonic CPC. The effects of the HO/CO system on the CPC development and function have not been studied.

The first cell in the SAN to produce an electrical impulse is not always the same (called the pacemaker shift). Normal sinus rhythm is the expected result. Pathophysiologic abnormalities relating to the SAN include sinus node dysfunction, sinus pause and arrest, SAN nodal block (first degree, second degree, and third degree), and sinus arrhythmia.^35^ Multiple investigators have suggested that the SAN has an intracellular Ca^2+^ clock which plays a critical role in impulse generation via RyR2. In a mouse model, Landstrom and colleagues^36^ determined that Jph2 negatively regulates RyR2-mediated calcium leak. The role of the HO/CO system on the intracellular Ca^2+^ clock has not been studied. In a mouse model, Mommersteeg and colleagues^37^ determined that NK2 homeobox 5 gene (Nkx2-5) is required to the gene expression border between the SAN and atrium. Nkx2-5 (a mesodermal cardiac transcription factor) activates gene expression for structural proteins, myosin heavy chain, troponin, and myosin light chanin-2, ventricular isoform (MLC-2v). The HO/CO system affects Nkx2-5 during embryonic stem cell differentiation into cardiomyocytes and result in contractile function.^38^ In an isolated preparation of murine myocardium, Abramochkin et al.^39^ suggested that the cardiac effects of CO may be associated with the activation of soluble guanylate cyclase with a resulting increase in cyclic adenosine monophosphate intracellularly. This leads to inhibition of phosphodiesterase 3, increased cyclic adenosine monophosphate, and positive chronotropy in the SAN. CO stimulates phosphodiesterase 2 in the contracting myocardium (enhances cyclic adenosine monophosphate degradation and produces AP shortening). Peters et al.^40^ evaluated electrophysiologic effects of cigarette smoking in patients with and without chronic beta-blocker therapy. Nicotine, catecholeamine, and CO increased. The authors concluded that a direct effect of nicotine or other components of tobacco smoke (including CO) may result in increased heart rate and improvement in atrioventricular conduction. Cardiac ganglia found around the SAN impact the node through the release of neurotransmitters and are affected by the HO system – heme oxygenase 2 (HO-2) (isoform of HO) may be a modulator of neurotransmission of cardiac ganglia and affect SAN function.^41^ Sick sinus syndrome (usually seen in older patients), however, is dependent on the Nrf-2/HO-1 pathway in a mouse model of sick sinus syndrome.^17^ Research regarding the effects of the HO/CO system on the SAN in humans is needed to better understand the role(s) of the system on the initiation and propagation of cardiac APs and possible HO/CO system therapies for electrophysiologic abnormalities.

## Heme Oxygenase/Carbon Monoxide System and Conduction in the Atrium

**Figure 7:**
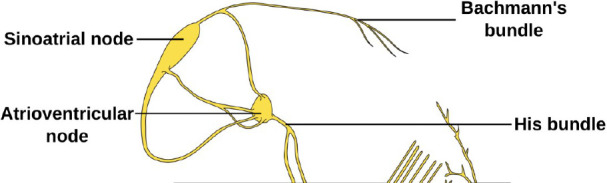
Special tracts from sinoatrial node to atrioventricular node. The electrical signal from the sinoatrial node to the atrioventricular node travels via the anterior, middle and posterior tracts – specialized internodal tracts within the atria. The tracts have specialized, Purkinje-like cells that propagate impulses faster than the surrounding atrial muscle. Madhero88; Angelito7, CC BY-SA 3.0, via Wikimedia Commons.

The AP that was created by the SAN spreads through internodal (interatrial) conduction tracts (specialized myocytes that lie between the SAN and AVN) and results in atrial contraction (**Figure 7**). Three discrete main pathways have been identified – anterior (leaves anterior surface of SAN, passes posteromedially to interatrial septum and then inferiorly, and splits into two bundles (one passes to left atrium, the other passes posterior to torus aorticus, finally reaching the AVN), middle (leaves the posterior and superior surface of SAN, passes inferiorly and medially around orifice of inferior vena cava, reaches interatrial septum where it passes inferiorly to the AVN), and posterior (leaves the posterior and inferior surface of the SAN, passes inferiorly through the crista terminalis, passes medially around valve of the inferior vena cava, reaches posterior surface of AVN).^42^ The cells appear to be similar to the Purkinje cells of the ventricles. While these internodal conduction tracts result in the transmission of the AP from the SAN to the AVN, intra-atrial conduction is necessary for atrial contraction. Conduction to the right atrium at its septum is from anterior and middle tracts and to the free wall involves conduction at the pectinate muscle from the terminal crest. Conduction to the left atrium is primarily through Bachmann’s bundles and their branches from anterior route (minor contributions are from middle internodal route) (**Figure 2**). Bachmann’s bundle is at the anterior aspect of the wall of left and right atrial chambers. Bachmann’s bundle affects rapid conduction of impulses from the right atrium to the left atrium. Other pathways, coronary sinus, anterior interatrial septum, and posterior interatrial septum, are also associated with interatrial conduction.^43^,^44^ The coronary sinus receives conduction from the posterior tract as well.^45^

Atrial cardiomyopathy (which can result in electrical and structural remodeling of the atria) is associated with electrical and contractile dysfunction.^46^ Electrical and structural remodeling of atria, response to external and internal stimuli, is a feature of atrial fibrillation and is affected by HO – a negative feedback regulation of HO-1 in activated atrial myocytes may offer protection against the development of atrial fibrillation and atrial fibrillation-related remodeling.^47^ Delays in the tracts can cause interatrial block (conduction delay between right and left atria), prolonged P wave (can be caused by delays in Bachmann’s bundle - muscular structure that connects the right and left atria of the heart), supraventricular arrhythmias, and/or asynchronous contraction.^48^ Studies of the HO/CO system on the function of these tracts have not been done and are needed to better understand the effects on travel of the AP from the SAN to the AVN, the coordinated contracture of the right and left atria, and possible use of the HO/CO system to treat diseases of these tracts and atrial contraction resulting from the AP’s.

## Heme Oxygenase/Carbon Monoxide System and Atrioventricular Node

The AVN is located in the triangle of Koch (boundaries are septal leaflet of tricuspid valve, ostium of coronary sinus, tendon of todaro, and tricuspid valve annulus) whose primary function is conducting atrial impulses from the atria to the ventricles. The structure is more organized than the SAN and usually provides a single conduction channel into the atrioventricular bundle (His bundle) and distal system. Its other functions include slowing of impulses, intrinsic pacemaker, electrical impedance, and delay.^49^,^50^ The distinct structural components of the AVN – compact node, lower nodal bundle, transitional tissue, left and right inferior nodal extension – are based on analysis of gap junction proteins and slow pathway conduction may be affected by the inferior nodal extension.^51^ An electrical gatekeeper that allows for ventricular filling, the AVN transmits electrical excitation originating in the SAN through slow or fast pathways to the ventricle.^52^ AVN delay is related to ion channel composition, intercellular communication, and its architecture. AVN pacemaker cells have neuron-like electrophysiological properties (i.e., refractoriness, excitability, automaticity, conductivity affected by excitatory, inhibitory, and modulatory neurotransmitters.)^52^ In a mouse model, Liang et al.^53^ determined that the γ-aminobutyric acidergic system contributes to the AVN delay as well. Slow and fast pathway electrophysiological characteristics are different and related to molecular composition and structure (i.e., gap junctions, ion channels, cellular/tissue geometry).^54^ Different cell types have different cellular electrophysiology that are associated with ion channel and connexin presence and cannot be identified on histology.^54^,^55^ Yanni et al.^56^ observed rings of conduction tissue in hearts of mice, guinea pigs, and rats that were negative for Cx43 and positive for hyperpolarization-activated cyclic nucleotide-gated channel 4 that originated from inferior extensions of the AVN, passed to the right and left to encircle the orifices of the mitral and tricuspid valve and reunited to form a retroaortic node.

The AVN filters atrial impulses, serves as a pacemaker, and results in half of the atrioventricular delay needed for cardiac output, which is determined by cellular automaticity, slow conduction, and long refractory period. The dual pathways impact its refractory and conduction properties.^54^ Electrophysiology of the AVN undergoes maturational changes from in utero to death. A dynamic structure, there are relative differences in AVN conductance and refractoriness in individuals aged 13 years and younger compared to adolescents compared to adults. Anterograde atrioventricular block is related to age; there is not significant difference in ventriculoatrial conduction related to age, antegrade conduction is better than retrograde conduction in children, and an increased incidence of dual atrioventricular pathways is seen in adolescents relative to children.^57^

Involved in several types of arrhythmias (atrioventricular reentrant tachycardia, Wolf–Parkinson–White syndrome, junctional ectopic tachycardia, atrial flutter, and atrial fibrillation), the AVN can protect the ventricles from atrial arrhythmias – slows impulses, can act as a secondary pacemaker, insulates the ventricles, and protects from atrial arrhythmias.^58^,^59^ Heterogeneity of gap junctions and ion channels results in AVN electrophysiological heterogeneity.^60^ Although studies of the physiologic effects of the HO/CO system on the AVN have not been done, the HO/CO system may interact with ion channels in the heart by increasing HO-1 with stress, heme-binding ion channels, decreasing reactive oxygen species production, and directly modulating T-type Ca^2+^ channels.^61^

Gap junctions allow direct communication between cells, affect the speed and conduction of electrical impulses through electrical coupling of cardiomyocytes. These low resistance pores, composed of connexins, result in cell type–specific, regional, and heart chamber–specific expression patterns which include specialized conduction tissues and endothelial cells.^62^ Composed of multiple connexins, cardiac myocytes adjust their level of coupling by modulation of channel properties, changes in connexin expression, and regulation of connexin turnover and trafficking.^63^ (Five different connexins are seen in heart muscle – Cx31.9, Cx37, Cx40, Cx43, and Cx45.) The functional role of the AVN is dependent on connexins and distribution varies in the different tissues.^64^ While strongly expressed in the early embryonic myocardium, Cx45 is dominant in the AVN and is crucial for electrical conduction in the node.^65^ In adult hearts, it is primarily restricted to the conduction system and optimally propagates impulse of the AVN and stabilizes the level of Cx30.2 in the adult mouse heart.^65^ The different connexins can combine to form different types of gap junction channels (heteromeric, heterotypic, homotypic). Two hemichannels (connexins, composed of six connexin protein subunits) connect cytoplasm of adjacent cells. The different types of channels have different permeabilities to ions and second messengers and different electrophysiologic properties. The transitional zone contains heterotypic Cx43/Cx45 that are sensitive to transjunctional voltage. Cx40, Cx43, and Cx45 are found in working and conductive myocardial cells. Cx30.2 forms gap junction channels with low sensitivity to transjunctional voltage and low unitary conductance.^65^,^66^ Studies on the relationship between connexins and HO are limited. Zhou and colleagues^67^ evaluated the role of Nrf2/HO-1/CO in mediating vincristine-induced neuroinflammation by inhibiting Cx43 production. Lakkisto et al.^68^ determined that pharmacological modulation of the HO-1 pathway may provide a new therapeutic approach to protect the heart against post-ischemic dysfunction and ischemia-induced ventricular fibrillation, possibly by Cx43-dependent mechanism. Further studies are needed to evaluate the effects of the HO/CO system on connexins as well as other biochemistries of the AVN.

## Heme Oxygenase/Carbon Monoxide System and Atrioventricular Bundle

The atrioventricular bundle, His bundle, has specialized muscle cells that transmit electrical impulses from the AVN to the ventricles. It extends from the AVN through the interventricular septum before splitting into the left and right bundle branches. The electrical path to transmit impulses is through the fibrous central body separating the atria and ventricles. Morphologic studies show that His bundle cells can be identified by their morphometric and histologic characteristics and allow differentiation from other cell types (i.e., nodal cells, Purkinje cells).^69^ The His bundle is composed of pacemaker cells, Purkinje cells, transition cells, and muscle cells. Insulated muscle fibers rapidly transmit electrical impulses distally.^70^ By transmitting electrical signals from the AVN to the ventricles, the His bundle synchronizes contractions and paces heart contractions.^71^,^72^ Earliest identification of His bundle in the chick model is at 5½ to 6 days and is derived from the low interatrial septum.^73^ A built-in pacemaker of the heart, the His bundle can generate a heart rate of 30 to 40 beats per minute.^74^ Its’ electrocardiography is helpful in diagnosing third degree block, localization of second degree blocks, first degree blocks, trifascular blocks, and Mobitz I and II blocks.^71^ His bundle pacing has been used for pacing-induced cardiomyopathy, bradycardia, narrow complex QRS, where rate-controlled atrial fibrillation leads to left ventricular failure, failed coronary sinus lead implantation, atrial fibrillation that is not adequately controlled and requires AVN ablation, and heart failure patients not eligible for cardiac resynchronization therapy.^75^,^76^,^77^

The biochemistry of the His bundle reflects the composition of the specialized muscle cells (well developed Golgi apparatus, unique intercalated discs, high concentration of mitochondria).^78^ Abnormalities can result in multiple arrhythmias. A region of the atrioventricular junction, activation of the His bundle results in engagement of left and right fascicles. His bundle pacing, more physiological than right ventricular apical pacing, targets the area of the septum that directly captures the conduction system.^77^ Junctional ectopic tachycardia (two forms – congenital and postoperative) is a rare arrhythmia associated with a high rate of morbidity and mortality and is due to automatic foci arising from the AVN and the His bundle area.^79^,^80^ Risk factors include a family history, body temperature, cardiopulmonary bypass, age, surgery, medications, and electrolyte abnormalities.^81^

Known effects of the HO/CO system on the heart include anti-inflammatory, cardioprotective, anti-apoptotic, and anti-oxidative properties and it modifies the function of mitochondria.^82^ The HO/CO system has not been studied in the His bundle system specifically. However, Jez et al.^83^ showed alterations in electrophysiological pathways in human induced pluripotent stem cells derived cardiomyocytes lacking HO-1. Complete atrioventricular block in mice results in arrhythmogenic cardiac remodeling. Duong and colleagues^84^ determined that sodium/calcium exchanger 1, regulated by miR-135a, affects arrhythmogenic Ca^2+^ loading/spontaneous Ca^2+^ release events, Ca^2+^ extrusion, and cardiomyocyte automaticity. miR-135a affects the expression of ion channels and transporters in the CCS and, possibly, affects spontaneous calcium release calcium efflux, and cardiac automaticity. Sodium/calcium exchanger 1, a sodium-calcium exchanger and involved in calcium homeostasis, may be affected by products of HO-1.^85^,^86^ Ablation of sodium/calcium exchanger 1 results in slowing of the heart rate and severe arrhythmias.^87^ Further evaluation of the HO/CO system on its effects on the biochemical properties of the His bundle is needed to further define these effects.

## Heme Oxygenase/Carbon Monoxide System and Bundle Branches

Subendocardial myocytes give rise to the bundle branches. These cells have a high expression of voltage-gated Na^+^ channels and connexin proteins which result in rapid conduction velocity.^88^ Arising from the His bundle, the left bundle branch serves the left ventricle with the right bundle branch serving the right. The former splits into the left anterior and left posterior fascicles and extend to the base of the papillary muscles and next to the myocardium.^89^ Left and right bundles are activated almost simultaneously. The left bundle branch usually activates the anterior interventricular septum first and results in left-to-right activation.^90^

Bundle branch block results in the right and left sides of the heart beating out of sync. A bundle branch block can affect right, left, or both ventricles.^91^,^92^ Effects can be minimal to marked reduction in ventricular function.^93^ Left bundle branch block can result in mechanical dysynchrony, left ventricle remodeling, and/or changes in the mitral valve apparatus.^94^ Treatment using physiologic pacing results in more physiologic activation of the ventricles.^95^,^96^ Left bundle branch pacing provides stability of the lead, treatment of conduction diseases, and pacing threshold is stable and low.^97^

Studies on the role(s) of the HO/CO system on the bundle branches have not been done and are needed. The toxicity of CO is known to result in delayed conduction through the bundle branches – reversible bundle branch block can occur.^98^ CO reduction of oxygen to the heart can affect electrical conduction of the entire electrical system of the heart, including the bundle branches. But the physiologic role(s) of the HO/CO system on the bundle branches have not been determined. Improved understanding of the normal effects of the HO/CO system on the bundle branches will help in treating pathologies associated with the bundle branches and result in approaches to normalizing cardiac function due to abnormalities of the bundle branches.

## Heme Oxygenase/Carbon Monoxide System and Peripheral Conduction System

Purkinje fibers, specialized cells that transmit electrical impulses to the ventricles, are located in the subendocardium of the ventricular walls.^99^ The Purkinje fibers have a different ion channel expression and conduction is faster than in the surrounding myocardium.^100^ Purkinje cell APs, longer than ventricular cells, show two levels of resting potential, transmit cardiac impulses rapidly to ventricular cells and have triggered and pacemaker activity^101^ (**Table 2**). Na^+^ channel isoforms are different between Purkinje cells and ventricular cells.^102^ Calcium currents are different as well – T-type being in Purkinje cells and L-type in ventricular cells.^103^ Transient outward currents of Purkinje cells differ from those of ventricular cells.^104^ Rapid delayed rectifier current (IKr) and slow delayed rectifier current (IKs) are smaller in Purkinje cells compared to ventricular cells.^105^ Na^+^/K^+^-ATPase is higher in ventricular tissues when compared to Purkinje cells.^106^ Overdrive suppression is present and is a result of Na/K pump activity.^107^ AP duration phenotype is different from that of ventricular cells and excitation coupling of the cell membrane with Ca^2+^ release in Purkinje cells is different from that of cardiomyocytes.^108^

**Table 2:** Differences in action potentials between Purkinje fibers and contractile cardiomyocytes

	Purkinje fibers	Contractile cardiomyocytes
Cellular function	Rapid, efficient electrical conduction	Produces force through muscle contraction
Conduction velocity	2–3 m/s	0.1–0.2 m/s
Action potential type	Fast-response, but with some automaticity	Fast-response
Upstroke (Phase 0)	Faster upstroke velocity (high expression of voltage-gated sodium channels	Fast upstroke velocity
Early repolarization (Phase 1)	More prominent, with a notch	Less prominent or absent
Plateau (Phase 2)	More negative plateau potential	Characteristic plateau phase, sustained longer for contraction
Repolarization (Phase 3)	Longer action potential duration at 90% repolarization	Rapid repolariazation
Diastolic period (Phase 4)	Slow diastolic depolarization (with small degree of automaticity	Stable resting membrane potential, no spontaneous depolarization
Action potential duration	Longer duration	Shorter duration

There are limited studies on the effects of the HO/CO system and cardiac Purkinje cells. Oxidative stress resulting in excess reactive oxygen species damages lipids and proteins, changes the function of ion channels, changes calcium handling, and results in changes in energy production in Purkinje cells.^109^,^110^,^111^ Protection of cardiac Purkinje cells by the HO/CO pathway is thought to be through its antioxidant and anti-inflammatory properties.^112^ Primarily through the production of CO, the HO/CO pathway is able to affect mitochondrial function and ion channel activity, resulting in changes in electrical signals in the heart.^113^,^114^ Effects of CO is dependent on its ability to interact with transition metals - CO readily binds to iron atoms in heme groups and can interfere with cellular signaling pathways dependent on the metal center.^115^,^116^ Nitric oxide (NO) formation also affects cellular effects of CO. NO formed in cardiac Na^+^ channels (form part of a macromolecular complex) can result in nitrosylation of the channel protein and induction of the late Na^+^ current.^117^ Arrythmias induced by CO are a result of stimulation of NO production by CO with a resultant increase in the late Na^+^ channel.^118^,^119^,^120^ This “late sodium current” (results in delayed “leak” of sodium ions into the cardiac cell) contributes to abnormal electrical activity and may result in arrhythmias when abnormally elevated, which affects the electrical stability.^121^ In the peripheral CCS, this permits a small sustained influx of sodium ions, which can contribute to the AP duration and influence heart rhythm, which can be a significant factor in certain arrhythmias.^113^ Future studies to better determine the role(s) of the HO/CO system on the peripheral conduction system are needed.

## Summary

The HO/CO system is crucial for normal growth, development, maturation, repair, and regeneration of cardiomyocytes. Studies on the effects of HO/CO on contractile cardiomyocytes are ongoing. While contractile and conduction cardiomyocytes originate from the same pool of progenitor cells during embryonic development (the CCS is formed by specialized cardiomyocytes), it is not clear if the HO/CO effects seen in the contractile cardiomyocytes are mirrored in the cells of the CCS. The complex cardiac electrical conduction system, consisting of cardiomyocyte cells that are specialized for conduction (SAN, AVN, His bundle, bundle branches, and peripheral conduction system), is affected negatively by toxic CO exposure. The lack of HO has been linked to abnormalities in the CCS, but the pathophysiology remains unclear. Effects under “normal” physiologic conditions have not been defined. HO mitigates oxidative stress, regulates inflammation, and maintains mitochondrial function in the cardiac electrical system, thereby helping to prevent arrhythmias and preserving normal cardiac electrical conduction.^115^ Evaluation of the HO/CO system on the conduction system has been limited. Studies that determine and define the role(s) of the HO/CO system on the electrical system of the heart are needed. Therapeutic effects must be evaluated as well.

Histologically, conduction cardiomyocytes are different from contractile cardiomyocytes (**Table 3**). The CCS heterogeneous structures are dynamic and variable. SAN (primary pacemaker of the heart) histology shows a collection of “nodal cells” in a connective tissue matrix with transitional cells at the borders where the “nodal cells” meet the atrial myocardium - there is a gradient of AP shape, pacemaking, connexin expression, Ca^2+^ handling, ionic current densities, cell size, and myofilament density. HO-1 regulates mitochondrial quality control in the SAN by regulating mitochondrial mitophagy, fusion, fission, and mitochondrial biogenesis.^122^ HO-1 production of CO impacts AVN activity by modulating ion activity within the node. Normal function of the heterogenous cells of CCS most likely is dependent on the HO/CO system, but little is known about the effects of the system on the various conduction components. Studies to evaluate the role of the HO/CO system on these different conduction components have not been done and are needed to define these functions. Knowledge of the effects of the HO/CO system during embryologic development of the CCS through death will allow us to affect pathological changes in the cardiac system during the human life cycle and, by doing so, impact clinical outcomes.

**Table 3:** Histological differences between cardiac conduction cardiomyocytes and contractile cardiomyocytes

	Cardiac conduction system	Contractile cardiomyocytes
Staining appearance	Pale, light-staining cytoplasm	Dark, distinct striations
Myofibril content	Few, poorly organized, most often located peripherally	Well-organized and abundant occupying most of the cytoplasm
Number of nuclei	Mono- or multi-nucleated (Purkinje fibers)	Usually mononucleated
Intercalated discs	Less prominent, fewer and smaller gap junctions in nodes	Prominent, abundant desmosomes and gap junctions
T-tubule system	Absent or poorly developed	Abundant, well-developed
Glycogen content	High	Lower
Cell size	Smaller (nodal cells), larger (Purkinje fibers)	Usually smaller than Purkinje fibers
Primary function	Initiates and conducted electrical impulses	Synchronizes and produces mechanical contraction

The cardiac conduction system consists of sinoatrial node, atrioventricular node, His bundle, bundle branches, and Purkinje fibers.

Studies are very limited with regards to the effects of the HO/CO system on the CCS. Therefore, there is a lack of effective data. While the HO/CO system is known to interact with multiple biochemical pathways and interactions vary during different stages of life and in different organisms, HO/CO system effects are complex and interactive. Studies to evaluate its roles in cardiomyocytes are ongoing. Therapies using the HO/CO system for pathologies of the contractile cardiomyocytes are being evaluated and developed. HO/CO system effects on the specialized cardiomyocytes of the developing CCS and its later stages of growth, development, and maturation may be similar to those of contractile cardiomyocytes, but current knowledge is at the basic level. Studies are needed to determine the specific roles of the HO/CO system on the specialized cells of the CCS to improve our understanding of the differences and similarities between the CCS and conduction cardiomyocytes. Directed treatments could then be developed for specific CCS pathologies associated, with the HO/CO system with the goal being improvement of cardiac function and health related to the CCS system.

## Data Availability

*Not applicable.*
